# The incidence of different forms of ileus following surgery for abdominal birth defects in infants: a systematic review with a meta-analysis method

**DOI:** 10.1515/iss-2020-0042

**Published:** 2021-08-17

**Authors:** Laurens D. Eeftinck Schattenkerk, Gijsbert D. Musters, David J. Nijssen, Wouter J. de Jonge, Ralph de Vries, L.W. Ernest van Heurn, Joep P.M. Derikx

**Affiliations:** Department of Paediatric Surgery, Emma Children’s Hospital, Amsterdam UMC, University of Amsterdam and Vrije Universiteit Amsterdam, Amsterdam, Netherlands; Tytgat Institute for Liver and Intestinal Research, Amsterdam UMC, University of Amsterdam, Amsterdam, Netherlands; Department of General, Visceral-, Thoracic and Vascular Surgery, University Hospital Bonn, Bonn, Germany; Medical Library, Vrije Universiteit, Amsterdam, Netherlands

**Keywords:** abdominal birth defect, adhesive small-bowel obstruction, anastomotic stenosis, ileus, infant, paediatric surgery

## Abstract

**Objectives:**

Ileus following surgery can arise in different forms namely as paralytic ileus, adhesive small bowel obstruction or as anastomotic stenosis. The incidences of these different forms of ileus are not well known after abdominal birth defect surgery in infants. Therefore, this review aims to estimate the incidence in general between abdominal birth defects.

**Content:**

Studies reporting on paralytic ileus, adhesive small bowel obstruction or anastomotic stenosis were considered eligible. PubMed and Embase were searched and risk of bias was assessed. Primary outcome was the incidence of complications. A meta-analysis was performed to pool the reported incidences in total and per birth defect separately.

**Summary:**

This study represents a total of 11,617 patients described in 152 studies of which 86 (56%) had a follow-up of at least half a year. Pooled proportions were calculated as follows; paralytic ileus: 0.07 (95%-CI, 0.05–0.11; *I* ^2^=71%, p≤0.01) ranging from 0.14 (95% CI: 0.08–0.23) in gastroschisis to 0.05 (95%-CI: 0.02–0.13) in omphalocele. Adhesive small bowel obstruction: 0.06 (95%-CI: 0.05–0.07; *I* ^2^=74%, p≤0.01) ranging from 0.11 (95% CI: 0.06–0.19) in malrotation to 0.03 (95% CI: 0.02–0.06) in anorectal malformations. Anastomotic stenosis after a month 0.04 (95%-CI: 0.03–0.06; *I* ^2^=59%, p=0.30) ranging from 0.08 (95% CI: 0.04–0.14) in gastroschisis to 0.02 (95% CI: 0.01–0.04) in duodenal obstruction. Anastomotic stenosis within a month 0.03 (95%-CI 0.01–0.10; *I* ^2^=81%, p=0.02) was reviewed without separate analysis per birth defect.

**Outlook:**

This review is the first to aggregate the known literature in order approximate the incidence of different forms of ileus for different abdominal birth defects. We showed these complications are common and the distribution varies between birth defects. Knowing which birth defects are most at risk can aid clinicians in taking prompt action, such as nasogastric tube placement, when an ileus is suspected. Future research should focus on the identification of risk factors and preventative measures. The incidences provided by this review can be used in those studies as a starting point for sample size calculations.

## Introduction

Ileus following surgery, consisting of both paralytic and mechanical causes, is a frequent complication after abdominal surgery, leading to increased morbidity, mortality, medical costs, and increased length of hospital stay [1], [2], [3], [4]. It is a clinical diagnosis which is characterized by intolerance to oral feeds, vomiting, abdominal distention, and the absence of flatus or stool.

Ileus following surgery can arise in different forms which depend on the definition used. Common causes are paralytic ileus, adhesive small bowel obstruction (SBO) and anastomotic stenosis. Paralytic ileus is a transient form of ileus which arises shortly after the operation in a response to surgical stress and is based on temporarily intestinal paralysis [5]. Anastomotic stenosis and SBO are mechanical forms of ileus that present later after surgery and could lead to reoperation.

Since abdominal birth defects are rare, the incidence of these forms of ileus are not well known. Yet, knowing the incidences would provide context for clinical decision making as well as a starting point for future research into preventative measures. Therefore, the objective of this review is to systematically aggregate the available data on the incidence of different forms of ileus following surgery for birth defects in infants.

## Methods

Studies were selected according to the criteria outlined below based on the PRISMA Guidelines [6]. Our protocol has been registered with the International Prospective Register of Systematic Reviews (PROSPERO) on 7 March 2019 (registration number: CRD42019119268).

### Participants

All studies reporting on any form of ileus following surgery for birth defects as primary of secondary endpoint were considered eligible. Only articles that described infants (≤three years) and specifically named the different forms of ileus were included. Animal studies, *in vitro* studies, non-English or non-Dutch articles, congress abstracts and studies with less than 10 cases were excluded.

### Search strategy

The electronic databases of the National Institutes of Health PubMed and EMBASE were systematically searched in February 2020 using both simple search terms as well as hierarchical family forms (e.g. MESH). The search strategy was designed together with a medical information specialist (RV). It combined four groups of search terms and their equivalents [1]: terms related to the age group of the patients at the moment of surgery (e.g. *infantile patients*) [2]; terms related to the location of surgery (e.g. *abdominal surgery*) [3]; terms related to congenital abdominal anomalies (e.g. *gastroschisis*) [4]; terms related to post-operative complications (e.g. *adhesive ileus*). Mesh and search terms used in Pubmed are included in Appendix 1.

### Primary and secondary outcomes

The primary endpoint was the pooled percentage of the three forms of ileus. We separately reviewed anastomotic stenosis within a month and after one month. Secondary endpoint was the pooled percentage per birth defect.

Terms included in paralytic ileus are: ileus (not related to anastomotic stricture) and post-operative ileus. Terms that only implied feeding problems without specifying the reason were not included. Terms included in adhesive small bowel obstruction are: intestinal obstruction, small bowel obstruction, adhesive ileus and stricture (not related to anastomosis). No additional terms or definitions were used for anastomotic stenosis; each article that specifically stated anastomotic stenosis was included. The early anastomotic stenosis, occurring within one month, were excluded for the analysis into late onset anastomotic stenosis.

For each complication separately, a Forest plot was created containing the estimated overall pooled proportion and the corresponding 95%-CIs. In each Forest plot, we also reported the pooled proportion and CIs per disease if [1]; at least three studies reported the specific complication in the disease or [2] if the total number of patients with the disease was ≥100 combined [3]; there was at least one event of a complication present in all studies on a specific disease combined. Birth defects that did not meet these criteria were present in the overall pooled proportion and reported as residuals. Follow-up was reported when relevant.

For all studies with multiple arms, data of both trial-arms were combined. If only one arm matched the inclusion criteria, the appropriate arm was used. Additionally extracted parameters were: author, country of conduct, year of publication, journal, study design, duration of follow-up, number of participants and type of birth defect.

### Data extraction

Titles and abstracts were screened to exclude nonrelated publications. Screening was done by two independent authors (LES, DN) using Rayyan. Disagreements were resolved by discussion between the two reviewers. If no consensus could be reached, a third specialist author was consulted (JD, GM). Then, the full texts of the remaining articles were read to determine eligibility for inclusion (LES, DN). If the full text was not found the authors were contacted. The reference lists of the included articles were cross checked to find additional articles.

### Validity and eligibility assessment

All included articles were assessed for the methodological quality and risk of bias. For cohort studies the Newcastle Ottawa quality assessment scale was used [7]. In randomized controlled trials this was done using the Jadad scoring system [8]. The assessment was done by LES and DN separately.

### Data synthesis

For each complication and each disease in a study, a weighted average of the logit proportions was determined by the use of the generic inverse variance method. The logit proportions were back transformed to the summary estimate and 95%-CIs were obtained in a summary proportion representing the pooled proportion of the form of ileus. Heterogeneity was assessed using the *I* ^2^ and *χ* ^2^ statistics. Analyses were performed with the use of R-studio version 3.6.1 (package “meta” (Schwarzer, 2007) and “metaprop” (Viachtbauer, 2010)). The random-effects model was used for interpretation. Heterogeneity was deemed significant if the pooled data’s p value was <0.05 or *χ* ^2^ statistics were ≥75. Heterogeneity was interpreted as small (*I* ^2^≤0.25), medium (*I* ^2^=0.25–0.50) or strong (*I* ^2^≥0.50), according to Higgins [9].

## Results

### Study characteristics

**Figure 1:**
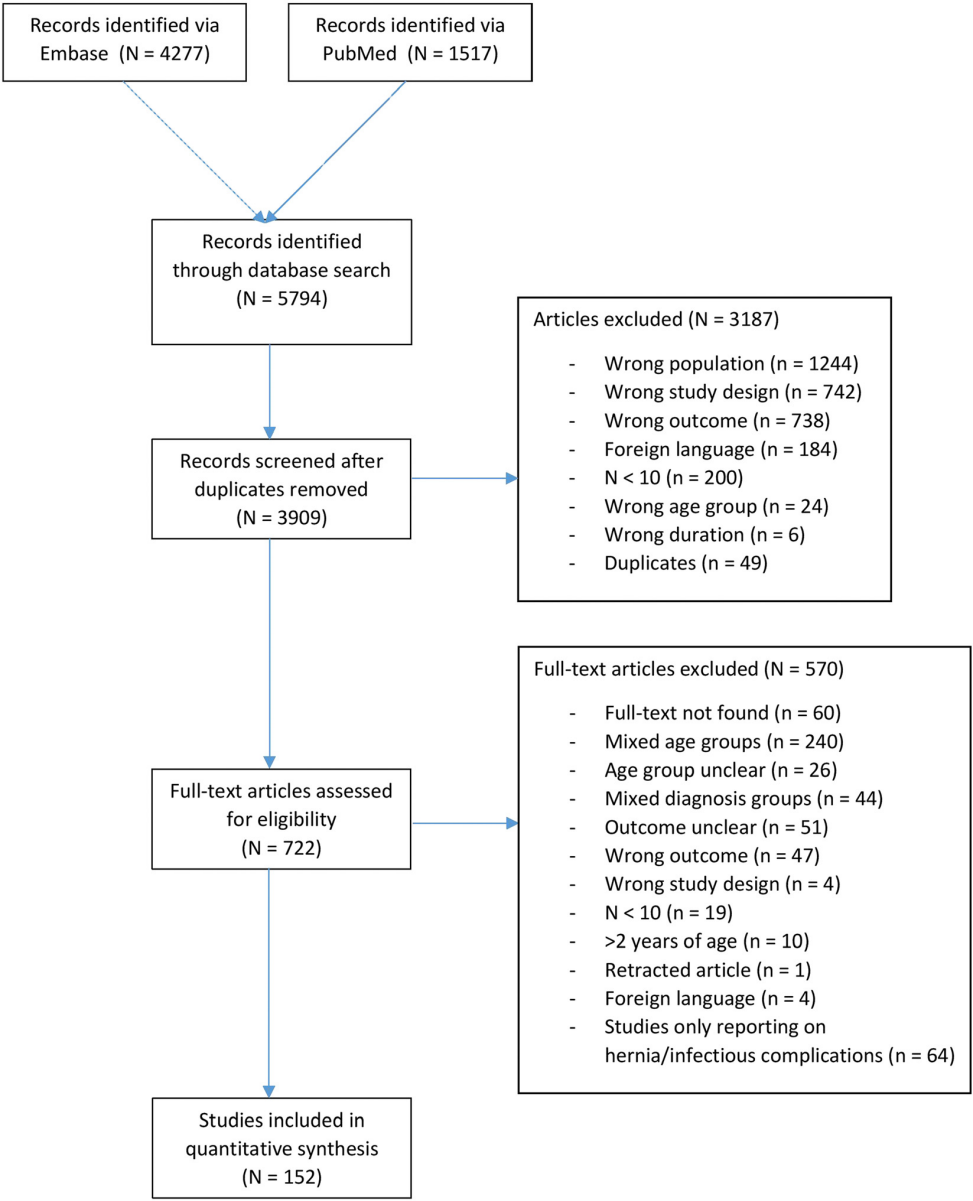
Flow-chart article selection.

In total, 5,784 records were identified. After automated removal of duplicates, 3,909 records were left for title and abstract screening. Of the 3,909 records, 722 were included and assessed for full text. Following full text evaluation 152 studies were included for quantitative analysis (Figure 1). Of the 152 studies, 118 were retrospective cohort studies, 25 were prospective cohort studies, four were retrospective multicentre cohort studies, two were retrospective matched case-control studies, two were randomized controlled trials and one was a combined study of a prospective and retrospective cohort. Studies were conducted in 31 different countries. Asian countries were most prominent with 58 studies, European studies represented 41, North- & South-America represented 38, Africa represented 9, The Middle-East 4 and Oceania represented 2. Of the 152 studies, 86 (57%) reported a follow-up of at least half a year.

This systematic review and meta-analysis represent 11,617 patients described in 152 studies presented in Table 1 []. Among these patients, the congenital conditions were divided as follows: Hirschsprung’s disease (n=4,341, 37%); gastroschisis (n=1,558, 13%); duodenal obstruction (n=1,068, 9%); anorectal malformations (n=1,047, 9%); small intestinal atresia (n=794, 7%); congenital diaphragmatic hernia (n=778, 7%); biliary atresia (n=681, 6%); malrotation (n=608, 5%); omphalocele (n=464, 4%); choledochal cyst (n=148, 1%); meconium ileus (n=54, >1%); Meckel’s diverticula (n=46, >1%); colonic atresia (n=30, >1%).

**Table 1:** Study characteristics.

Author	Year	Journal	Country	Design	FU>0.5y	Study duration	Anomaly	POI	SBO	AS < month	ÀS > month
Stollman	2008	Journal of Paediatric Surgery	Netherlands	Retrospective cohort study	No	1971–2004	Small intestinal atresia	2/110	12/110	1/110	6/110
Guo	2010	Transplantation Proceedings	China	Retrospective cohort study	No	2006–2009	Biliary atresia	2/22	X	X	X
Walter-Nicolet	2009	Journal of Paediatric Gastroenterology and Nutrition	France	Prospective cohort study	Yes	2004–2006	Gastroschisis	X	2/73	X	X
Wang	2013	Hepatobiliary Pancreat Dis Int	China	Retrospective cohort study	Yes	2008–2011	Biliary atresia	X	2/73	X	X
Lee	2012	Paediatric Surgery International	South Korea	Retrospective cohort study	No	2001–2010	Small intestinal atresia	X	3/11	X	X
Cox	2005	Paediatric Surgery International	South Africa	Retrospective cohort study	Yes	1966–2004	Colonic atresia	X	0/14	2/14	0/14
Festen	2002	Journal of Paediatric Surgery	Netherlands	Multi-centre (5) retrospective cohort	Yes	1980–1992	Small intestinal atresia	1/15	X	4/15	1/14
Escobar	2005	Journal of Paediatric Surgery	USA	Retrospective cohort study	Yes	1972–2004	Hirschsprung’s disease	X	5/33	X	5/33
Yan	2017	Biomedical Research	China	Prospective cohort study	Yes	2011–2014	Hirschsprung’s disease	2/38	2/38	X	2/38
Mirshemirani	2007	Acta Medica Iranica	Iran	Prospective cohort study	Yes	1993–2003	ARM	X	X	0/30	0/30
Dariel	2015	European Journal of Paediatric Surgery	Canada	Retrospective cohort study	Yes	2006–2010	Gastroschisis	X	6/63	X	X
Mendez-Martinez	2016	European Journal of General Medicine	Mexico	Prospective cohort study	No	2008–2013	Gastroschisis	6/42	7/42	X	X
Fredriksson	2015	British Journal of Surgery	Sweden	Retrospective cohort study	Yes	1976–2011	Hirschsprung’s disease	X	19/65	X	X
Fredriksson	2015	British Journal of Surgery	Sweden	Retrospective cohort study	Yes	1976–2011	Malrotation	X	13/45	X	X
Fredriksson	2015	British Journal of Surgery	Sweden	Retrospective cohort study	Yes	1976–2011	Small intestinal atresia	X	11/40	X	X
Fredriksson	2015	British Journal of Surgery	Sweden	Retrospective cohort study	Yes	1976–2011	Gastroschisis	X	9/85	X	X
Fredriksson	2015	British Journal of Surgery	Sweden	Retrospective cohort study	Yes	1976–2011	Duodenal obstruction	X	10/93	X	X
Fredriksson	2015	British Journal of Surgery	Sweden	Retrospective cohort study	Yes	1976–2011	Omphalocele	X	4/44	X	X
Fredriksson	2015	British Journal of Surgery	Sweden	Retrospective cohort study	Yes	1976–2011	Congenital diaphragmatic hernia	X	6/75	X	X
Fredriksson	2015	British Journal of Surgery	Sweden	Retrospective cohort study	Yes	1976–2011	ARM	X	4/58	X	X
Fredriksson	2015	British Journal of Surgery	Sweden	Retrospective cohort study	Yes	1976–2011	Biliary atresia	X	0/28	X	X
Werbeck	2010	Journal of Paediatric Surgery	USA	Retrospective cohort study	Yes	1991–2010	Gastroschisis	X	2/13	X	X
Demirogullari	2011	Paediatric Surgery International	Turkey	Retrospective cohort study	No	1998–2011	ARM	X	2/112	X	X
Rouzrokh	2010	Paediatric Surgery International	Iran	Retrospective cohort study	Yes	2006–2009	Hirschsprung’s disease	X	X	X	12/86
S. Li	2017	European Review for Medical and Pharmacological Sciences	China	Retrospective cohort study	Yes	2006–2013	Hirschsprung’s disease	X	X	0/15	X
Ghosh	2016	ANZ Journal of Surgery	Australia	Retrospective cohort study	Yes	2005–2012	Hirschsprung’s disease	X	3/50	X	2/50
Chen	2014	World J. Paediatric Surgery	China	Retrospective cohort study	Yes	2003–2012	Duodenal obstruction	X	4/287	X	X
Bianchi	1998	Seminars in Paediatric Surgery	England	Retrospective cohort study	Yes	1984–1997	Hirschsprung’s disease	X	2/13	X	X
Mattioli	1998	Journal of Paediatric Surgery	Italy	Retrospective cohort study	No	1993–1996	Hirschsprung’s disease	X	X	0/8	0/8
Teitelbaum	1998	Seminars in Paediatric Surgery	USA	Retrospective cohort study	Yes	X	Hirschsprung’s disease	X	4/24	X	0/24
Demirbilek	1999	Paediatric Surgery International	Turkey	Retrospective cohort study	Yes	1987–1997	ARM	X	X	X	1/31
Santos	1999	Journal of Paediatric Surgery	USA	Retrospective cohort study	No	1988–1999	Hirschsprung’s disease	X	4/65	X	X
de la Torre	2000	Journal of Paediatric Surgery	Mexico	Retrospective cohort study	Yes	1994–2000	Hirschsprung’s disease	X	0/10	X	X
Fleet	2000	Journal of Paediatric Surgery	England	Retrospective cohort study	Yes	1991–1997	Gastroschisis	X	X	X	1/10
Fleet	2000	Journal of Paediatric Surgery	England	Retrospective cohort study	Yes	1991–1997	Small intestinal atresia	X	X	X	1/6
Fleet	2000	Journal of Paediatric Surgery	England	Retrospective cohort study	Yes	1991–1997	Colonic atresia	X	X	X	0/3
Hay	2000	Journal of Paediatric Surgery	Egypt	Retrospective cohort study	No	X	Biliary atresia	X	1/21	X	X
Langer	2000	Journal of Paediatric Surgery	USA	Prospective cohort study	No	X	Hirschsprung’s disease	X	2/22	10/22	X
Patwardhan	2001	Journal of Paediatric Surgery	England	Retrospective cohort study	Yes	1994–1999	ARM	X	5/49	X	X
Snyder	2001	Journal of Paediatric Surgery	USA	Retrospective cohort study	No	1969–2000	Gastroschisis	X	21/199	X	X
Snyder	2001	Journal of Paediatric Surgery	USA	Retrospective cohort study	No	1969–2000	Small intestinal atresia	X	9/25	X	X
Höllwarth	2002	Journal of Paediatric Surgery	Austria	Retrospective cohort study	Yes	1988–2000	Hirschsprung’s disease	X	X	X	X
Saxena	2001	The world journal of Hernia	Germany	Prospective cohort study	Yes	1984–1998	Omphalocele	1/50	2/50	X	X
Saxena	2001	Paediatric Surgery International	Germany	Prospective cohort study	Yes	1984–1998	Gastroschisis	X	10/70	X	X
Önen	2003	Paediatric Surgery International	Turkey	Prospective/retrospective cohort	No	1990–2000	Meckel’s diverticulum	X	2/34	X	X
Shah	2003	Journal of Paediatric Surgery	India	Prospective cohort study	Yes	X	ARM	X	X	X	0/12
Weidner	2003	Journal of Paediatric Surgery	USA	Retrospective cohort study	No	1998–2001	Hirschsprung’s disease	X	0/15	X	X
Escobar	2004	Journal of Paediatric Surgery	USA	Retrospective cohort study	Yes	1972–2001	Duodenal obstruction	X	5/169	X	1/169
Kubota	2004	Journal of Paediatric Surgery	Japan	Prospective cohort study	Yes	1990–2001	Hirschsprung’s disease	X	1/41	X	X
Wester	2004	Journal of Paediatric Surgery	Finland	Retrospective cohort study	No	2000–2003	Hirschsprung’s disease	X	X	0/15	0/15
Majid	2015	Pakistan Paediatric Journal	Pakistan	Prospective cohort study	Yes	X	Duodenal obstruction	X	3/27	X	X
Sauer	2005	Journal of Paediatric Surgery	Canada	Retrospective cohort study	No	1999–2003	Hirschsprung’s disease	X	2/24	X	0/24
Thepcharoennirund	2005	Journal of Medical Association of Thailand	Thailand	Retrospective cohort study	No	1987–2004	Gastroschisis	12/129	X	X	X
Chiu	2006	Journal of Perinatal Medicine	USA	Retrospective cohort study	No	1994–2004	Gastroschisis	X	1/43	X	X
Choudhry	2006	Paediatric Surgery International	England	Retrospective cohort study	Yes	1998–2003	Gastroschisis	X	2/32	X	X
Choudhry	2006	Paediatric Surgery International	England	Retrospective cohort study	Yes	1999–2003	ARM	X	0/46	X	X
Choudhry	2006	Paediatric Surgery International	England	Retrospective cohort study	Yes	2000–2003	Omphalocele	X	0/25	X	X
Choudhry	2006	Paediatric Surgery International	England	Retrospective cohort study	Yes	2001–2003	Meconium ileus	X	5/20	X	X
Choudhry	2006	Paediatric Surgery International	England	Retrospective cohort study	Yes	2001–2003	Malrotation	X	3/23	X	X
Choudhry	2006	Paediatric Surgery International	England	Retrospective cohort study	Yes	2001–2003	Diaphragmatic hernia	X	1/25	X	X
Choudhry	2006	Paediatric Surgery International	England	Retrospective cohort study	Yes	2001–2003	Hirschsprung’s disease	X	2/33	X	X
Choudhry	2006	Paediatric Surgery International	England	Retrospective cohort study	Yes	2001–2003	Small intestinal atresia	X	4/36	X	X
Lee	2006	Journal of Paediatric Surgery	USA	Retrospective cohort study	Yes	1981–2002	Omphalocele	1/20	X	X	X
A. Li	2006	Chinese Medical Journal	China	Retrospective cohort study	Yes	1999–2004	Hirschsprung’s disease	X	1/252	X	16/252
Liem	2006	Asian Journal of Surgery	Vietnam	Prospective cohort study	Yes	2002–2004	Hirschsprung’s disease	0/53	X	X	X
Maksoud-Filho	2006	Paediatric Surgery International	Brazil	Retrospective cohort study	No	1998–2005	Gastroschisis	X	1/43	X	X
Owen	2006	Journal of Paediatric Surgery	England	Retrospective cohort study	No	1990–2004	Gastroschisis	X	0/48	X	X
Banieghbal	2007	Journal of Paediatric Surgery	South Africa	Prospective cohort study	Yes	2002–2005	Small intestinal atresia	X	X	2/16	X
Dutta	2007	Journal of laparoendoscopic	USA	Retrospective cohort study	No	2002–2005	Biliary atresia	X	2/10	X	X
Menon	2007	Journal of Paediatric Surgery	India	Prospective cohort study	Yes	1997–2005	ARM	X	X	X	0/46
Riehle	2007	Journal of Paediatric Surgery	USA	Retrospective cohort study	No	1993–2004	Congenital diaphragmatic hernia	X	7/125	X	X
Stringer	2007	Journal of Paediatric Surgery	United Kingdom	Prospective cohort study	Yes	1994–2006	Biliary atresia	X	2/60	X	X
Baglaj	2007	European Journal of Paediatric Surgery	United Kingdom	Retrospective cohort study	No	1986–2006	Small intestinal atresia	X	X	X	2/26
Henrich	2007	Paediatric Surgery International	Germany	Prospective cohort study	No	1994–2004	Gastroschisis	11/40	X	X	X
Henrich	2007	Paediatric Surgery International	Germany	Prospective cohort study	No	1994–2004	Omphalocele	3/26	X	X	X
Ishikawa	2008	Paediatric Surgery International	Japan	Retrospective cohort study	No	1990–2001	Hirschsprung’s disease	X	1/49	X	X
Shinall	2008	Journal of Paediatric Surgery	USA	Retrospective cohort study	No	1997–2001	Hirschsprung’s disease	X	X	X	3/60
Spilde	2008	Journal of Paediatric Surgery	USA	Retrospective cohort study	No	2003–2007	Duodenal obstruction	X	X	2/29	X
Tongsin	2008	Journal of Medical Association of Thailand	Thailand	Retrospective cohort study	No	1988–2007	Small intestinal atresia	9/142	3/142	X	8/142
van Eijck	2008	Journal of Paediatric Surgery	Netherlands	Retrospective cohort study	No	1971–2004	Gastroschisis	X	14/55	X	X
van Eijck	2008	Journal of Paediatric Surgery	Netherlands	Retrospective cohort study	No	1971–2004	Omphalocele	X	12/92	X	X
Zheng	2008	Paediatric Surgery International	China	Retrospective cohort study	No	2004–2007	ARM	X	1/38	X	X
Dassinger	2009	Paediatric Surgery International	USA	Retrospective cohort study	No	1993–2008	Colonic atresia	X	X	0/12	X
Ferreira	2009	Surgical Endoscopy	France	Prospective cohort study	No	X	Congenital diaphragmatic hernia	1/30	X	X	X
Gourlay	2008	Journal of Paediatric Surgery	USA	Retrospective cohort study	Yes	1993–2003	Congenital diaphragmatic hernia	X	4/38	X	X
Hua	2009	Ghang Gung Medical Journal	Taiwan	Retrospective cohort study	Yes	1991–2006	Choledochal cyst	X	1/30	X	X
Liu	2009	Journal of Laparoendoscopic	China	Retrospective cohort study	Yes	2003–2007	Biliary atresia	X	0/10	X	X
Obermayr	2008	European Journal of Paediatric Surgery	Germany	Retrospective cohort study	Yes	2002–2007	Hirschsprung’s disease	X	X	X	1/22
Takahashi	2009	Journal of Paediatric Surgery	Japan	Retrospective cohort study	Yes	1963–2008	Biliary atresia	1/12	X	X	X
Gunnarsdottir	2009	European Journal of paediatric Surgery	Sweden	Prospective cohort study	Yes	2000–2007	Hirschsprung’s disease	X	1/29	X	X
Hong	2010	European journal of Obstetrics & Gynaecology	China	Prospective cohort study	Yes	2004–2008	Gastroschisis	1/17	1/17	X	X
Payne	2010	Journal of Neonatal-Perinatal Medicine	USA	Matched case-control study	Yes	1999–2007	Gastroschisis	X	9/127	X	X
Vu	2010	Paediatric surgery International	Vietnam	Prospective cohort study	Yes	2004–2009	Hirschsprung’s disease	X	X	X	4/51
de Vos	2011	South African Journal of Science	South Africa	Retrospective cohort study	Yes	2000–2009	ARM	X	X	X	4/39
Hill	2011	Journal of Laparoendoscopic	USA	Retrospective cohort study	No	2001–2010	Duodenal obstruction	14/58	X	X	0/58
Karimi	2011	Paediatric Surgery International	Netherlands	Retrospective cohort study	No	1984–2007	Meconium ileus	X	4/34	X	X
Kozlov	2010	European Journal of Surgery	Russia	Retrospective cohort study	No	2005–2009	Duodenal obstruction	X	X	0/27	X
Travassos	2011	Journal of Paediatric Surgery	Netherlands	Retrospective cohort study	Yes	1988–2010	Hirschsprung’s disease	X	1/15	X	0/15
van der Zee	2011	World J. Surgery	Netherlands	Retrospective cohort study	Yes	2000–2010	Duodenal obstruction	X	X	X	1/28
Li	2012	Paediatric surgery International	China	Retrospective cohort study	No	2009–2012	Small intestinal atresia	X	3/35	X	X
Liem	2012	Journal of Paediatric Surgery	Vietnam	Prospective cohort study	Yes	2008–2010	ARM	X	X	X	0/10
Romao	2012	Journal of Paediatric Surgery	Canada	Retrospective cohort study	Yes	2000–2009	Congenital diaphragmatic hernia	X	3/22	X	X
Sato	2012	Paediatric Surgery International	Japan	Retrospective cohort study	No	2005–2011	Small intestinal atresia	1/25	X	X	X
Sato	2012	Paediatric Surgery International	Japan	Retrospective cohort study	No	2005–2011	ARM	1/13	X	X	X
Weil	2011	Journal of Paediatric Surgery	USA	Retrospective cohort study	No	2000–2009	Gastroschisis	X	X	X	X
Ghaffarpour	2013	Journal of Paediatric Surgery	Sweden	Retrospective cohort study	No	X	Duodenal obstruction	X	0/28	X	X
Ferreira	2013	Journal of Paediatric Surgery	France	Retrospective cohort study	Yes	2006–2010	Congenital diaphragmatic hernia	X	9/37	X	X
Jensen	2013	Journal of Laparoendoscopic	USA	Retrospective cohort study	No	2005–2011	Duodenal obstruction	X	X	X	1/66
Nam	2013	World Journal of Surgery	South Korea	Retrospective cohort study	Yes	2008–2011	Congenital diaphragmatic hernia	X	5/50	X	X
Nio	2013	Paediatric Surgery International	Japan	Prospective RCT	Yes	2006–2011	Biliary atresia	2/69	X	X	X
van der Zee	2013	World Journal of Surgery	Netherlands	Retrospective cohort study	Yes	2000–2011	ARM	X	1/19	X	1/19
Diao	2014	International Journal of Surgery	China	Retrospective cohort study	Yes	2011–2013	Choledochal cyst	X	0/27	X	X
Diao	2014	Journal of Paediatric Surgery	China	Retrospective cohort study	Yes	2011–2012	ARM	X	X	X	0/31
Elder	2014	Journal of Paediatric Surgery	USA	Retrospective cohort study	Yes	2000–2011	Malrotation	X	13/102	X	X
Friedmacher	2014	Paediatric surgery International	Austria	Retrospective cohort study	Yes	1975–2008	Gastroschisis	X	27/108	X	8/108
Ming	2014	Journal of Paediatric Surgery	China	Retrospective cohort study	Yes	1992–2012	ARM	X	X	X	3/66
Nasr	2014	Journal of Paediatric Surgery	Canada	Retrospective matched case control cohort study	No	2000–2010	Hirschsprung’s disease	15/54	1/54	X	4/54
Shrestha	2014	Journal Nepal Paediatric Society	Nepal	Prospective cohort study	Yes	2008–2013	Hirschsprung’s disease	X	0/12	X	0/12
Sulkowski	2014	Journal of Paediatric Surgery	USA	Retrospective multi-centre database research	Yes	1999–2009	Hirschsprung’s disease	X	82/1,555	X	83/1,555
Yang	2014	Journal of Paediatric Surgery	China	Retrospective cohort study	No	2011–2013	ARM	X	X	X	0/20
Madadi-Sanjani	2015	Biomedical Research International	Germany	Retrospective cohort study	No	1975–2008	Biliary atresia	X	5/153	X	X
Martinez-Criado	2015	Cirugia Espanola	Spain	Retrospective cohort study	No	2003–2012	Hirschsprung’s disease	X	6/73	X	4/73
Miyano	2015	Journal of Laparoendoscopic	Japan	Retrospective cohort study	Yes	2007–2012	Malrotation	X	1/14	X	X
Shangjie	2014	Cell Biochemical Biophysiology	China	Prospective cohort study	No	2009–2014	Hirschsprung’s disease	28/281	27/281	X	X
Almosallam	2016	Ann Saudi Medicine	Saudi Arabia	Retrospective cohort study	No	2000–2014	ARM	X	4/104	X	X
Diao	2016	Surgical Endoscopy	China	Retrospective cohort study	No	2013–2014	ARM	X	X	X	X
Guerra	2016	Journal of Paediatric Surgery	Canada	Retrospective cohort study	No	1995–2014	Hirschsprung’s disease	X	X	X	5/36
Inoue	2016	Surgical endoscopy	Japan	Retrospective cohort study	Yes	2000–2014	Congenital diaphragmatic hernia	X	4/24	X	X
Matsumoto	2016	Surgical Today	Japan	Retrospective cohort study	No	1997–2015	Choledochal cyst	X	2/13	X	X
Raitio	2016	European Journal of Paediatric Surgery	England	Retrospective cohort study	Yes	2002–2014	Malrotation	X	X	X	X
Diao	2017	Surgical endoscopy	China	Retrospective cohort study	No	2013–2016	ARM	X	X	X	0/15
Dingemann	2017	European Journal of Paediatric Surgery	Germany	Multicentre retrospective cohort	No	2007–2012	Omphalocele	X	1/54	X	X
C. Lu	2017	Journal of Paediatric Surgery	China	Multicentre retrospective cohort	Yes	2005–2012	Hirschsprung’s disease	X	X	X	21/650
Y. Lu	2017	Transplantation Proceedings	China	Retrospective cohort study	No	2009–2014	Biliary atresia	X	X	X	X
Oh	2017	Surgical Endoscopy	South Korea	Retrospective cohort study	No	2005–2015	Duodenal obstruction	X	X	X	0/22
Risby	2017	Journal of Paediatric Surgery	Denmark	Retrospective cohort study	Yes	1997–2009	Gastroschisis	X	12/47	X	X
Son	2017	Journal of Paediatric Surgery	Vietnam	Retrospective cohort study	No	2009–2015	Duodenal obstruction	X	X	X	2/112
Tyson	2017	Journal of Laparoendoscopic	USA	Retrospective cohort study	No	2007–2015	Congenital diaphragmatic hernia	X	5/54	X	X
van den Eijnden	2017	World Journal of Surgery	Netherlands	Retrospective cohort study	No	1989–2014	Choledochal cyst	X	1/30	0/30	X
Zani	2017	Paediatric Surgery International	Canada	Retrospective cohort study	Yes	2004–2014	Duodenal obstruction	X	4/92	X	6/92
Zmora	2016	American Journal of Surgery	USA	Retrospective cohort study	No	2007–2015	Gastroschisis	X	1/11	X	X
Zmora	2016	American Journal of Surgery	USA	Retrospective cohort study	No	2007–2015	Omphalocele	X	0/6	X	X
Avci	2018	Eastern Journal of Medicine	Turkey	Retrospective cohort study	No	2008–2017	Duodenal obstruction	X	2/32	X	X
Peng	2018	Journal of Paediatric Surgery	China	Retrospective cohort study	No	2011–2015	Small intestinal atresia	X	2/41	X	X
Xiao	2018	Medicine	China	Prospective cohort study	Yes	2011–2014	ARM	X	X	X	0/56
Xiao	2018	Journal of Surgical Research	China	Prospective RCT	Yes	2011–2015	Biliary atresia	X	5/166	X	X
Zhang	2018	Journal of Paediatric Surgery	China	Retrospective cohort study	Yes	2011–2014	Hirschsprung’s disease	X	X	X	0/23
England	2012	Journal of Paediatric Surgery	South Africa	Retrospective cohort study	Yes	2005–2009	ARM	X	X	X	12/42
Wakhlu	2000	Journal of Paediatric Surgery	India	Retrospective cohort study	Yes	1972–1998	Omphalocele	X	2/64	X	X
Pratap	2007	Journal of Paediatric Surgery	India	Retrospective cohort study	Yes	2002–2006	Hirschsprung’s disease	X	X	X	x
Abbas	2016	Journal of Paediatric Surgery	USA	Retrospective cohort study	Yes	2002–2015	Malrotation	X	6/56	X	X
Chan	2019	Journal of laparoendoscopic	Hong Kong	Retrospective database	Yes	1993–2007	Biliary atresia	X	2/22	X	X
de Bie	2019	Journal of Paediatric Surgery	Belgium	Retrospective multicentre database	Yes	2000–2016	Congenital diaphragmatic hernia	X	1/62	X	X
Dewberry	2019	Journal of Paediatric Surgery	USA	Retrospective cohort	No	2008–2018	Congenital diaphragmatic hernia	X	5/70	X	X
Dewberry	2019	Journal of surgical research	USA	Retrospective cohort	Yes	2007–2017	Small intestinal atresia	X	2/47	X	2/47
Dübbers	2002	European Journal of Paediatric Surgery	Germany	Retrospective cohort	Yes	1990–2000	Hirschsprung’s disease	X	2/35	X	X
Gabler	2018	South African Medical Journal	South Africa	Retrospective cohort	No	X	ARM	X	0/50	X	X
Gao	2019	Journal of International Medical Research	China	Retrospective cohort	No	2018	Meckel’s diverticulum	X	0/12	X	X
Gao	2019	Journal of International Medical Research	China	Retrospective cohort	No	2018	Hirschsprung’s disease	X	0/35	X	X
He	2016	Journal of Laparoendoscopic	China	Retrospective cohort	Yes	2011–2016	Congenital diaphragmatic hernia	X	1/14	X	X
Joda	2019	Updates in Surgery	Iraq	Prospective cohort	Yes	2010–2017	Small intestinal atresia	X	3/34	X	4/34
Jona	2001	Paediatric Endo-Surgery & Innovative Techniques	USA	Retrospective cohort	No	1993–2000	Hirschsprung’s disease	X	0/44	X	X
Long	2019	Arch Dis Child Fetal Neonatal Ed	England	Retrospective national database cohort	Yes	2009–2010	Congenital diaphragmatic hernia	X	9/140	X	X
Marei	2019	Egyptian Paediatric Association Gazette	Egypt	Retrospective cohort	Yes	2014–2017	Small intestinal atresia	3/22	X	1/22	X
Narang	2019	Journal of Obstetrics & Gynaecology	New Zealand	Retrospective cohort study	Yes	2011–2016	Gastroschisis	X	6/71	X	X
Narang	2019	Journal of Obstetrics & Gynaecology	New Zealand	Retrospective cohort study	Yes	2011–2016	Omphalocele	X	0/22	X	X
Jung	1995	Journal of Paediatric Surgery	South Korea	Retrospective cohort study	No	1980–1991	Hirschsprung’s disease	X	2/77	X	X
Ren	2018	Journal of Laparoendoscopic	China	Retrospective cohort study	Yes	2005–2016	ARM	1/25	X	X	X
Ryu	2019	Annals of Surgery Treatment and Research	South Korea	Retrospective cohort study	No	2001–2018	Choledochal cyst	X	1/43	X	X
Sakaguchi	2019	World journal of Paediatric Surgery	Japan	Retrospective cohort study	No	1995–2004	ARM	3/39	X	X	X
Sato	1998	Journal of Paediatric Surgery	Japan	Retrospective cohort study	No	1970–1997	Small intestinal atresia	X	2/88	X	2/88
Sola	2018	Paediatric Surgery International	USA	Retrospective cohort study	No	1999–2016	Hirschsprung’s disease	X	4/100	X	X
Yang	2019	Medicine	China	Retrospective cohort study	Yes	2013–2016	Small intestinal atresia	X	2/42	X	3/42
H. Zhu	2019	Journal of Paediatric Surgery	China	Retrospective cohort study	Yes	2008–2017	Small intestinal atresia	X	4/39	X	3/39
H. Zhu	2019	Paediatric Surgery International	China	Retrospective cohort study	Yes	2003–2017	Malrotation	X	10/252	X	X
T. Zhu	2019	International Journal of Colorectal Disease	China	Retrospective cohort study	Yes	2010–2015	Hirschsprung’s disease	X	X	X	0/157

Risk of bias was assessed and is shown in Table 2. Most studies included reported fair quality on the NOS which was also the case for the two RCTs using the Jadad score. The mean scores on the NOS of articles describing paralytic ileus and early anastomotic stenosis was slightly lower (5, 5) compared to articles describing adhesive small bowel obstruction and late onset anastomotic stenosis [6].

**Table 2:** Assessment risk of bias.

Author	Year	Jadad	New Ottawa scale (NOS)			
		Score	Selection	Comparability	Outcome	Total
		(0–5)	(0–4*)	(0–2*)	(0–3*)	(0–9)
Stollman	2008		***	*	***	7
Guo	2010		***	*	**	6
Walter-Nicolet	2009		***	**	***	8
Wang	2013		***	*	***	7
Lee	2012		***	*	**	6
Cox	2005		***	–	***	6
Festen	2002		**	**	**	6
Escobar	2005		***	–	***	6
Yan	2017		***	**	***	8
Mirshemirani	2007		***	–	***	6
Dariel	2015		***	*	***	7
Mendez-Martinez	2016		***	*	**	6
Fredriksson	2015		***	–	***	6
Werbeck	2010		**	–	**	4
Demirogullari	2011		**	–	**	4
Rouzrokh	2010		***	*	***	7
S. Li	2017		**	*	***	6
Ghosh	2016		**	*	***	6
Chen	2014		***	**	***	8
Bianchi	1998		***	–	***	6
Mattioli	1998		**	–	**	4
Teitelbaum	1998		**	–	***	5
Demirbilek	1999		***	–	***	6
Santos	1999		***	–	***	6
de la Torre	2000		***	–	***	6
Fleet	2000		**	*	***	6
Hay	2000		***	–	***	6
Langer	2000		***	*	**	6
Patwardhan	2001		***	*	**	6
Snyder	2001		***	**	**	7
Höllwarth	2002		***	–	***	6
Saxena	2001		***	*	***	7
Önen	2003		***	–	**	5
Shah	2003		**	–	***	5
Weidner	2003		**	**	**	6
Escobar	2004		***	*	**	6
Kubota	2004		**	*	**	5
Wester	2004		***	–	**	5
Majid	2015		***	**	***	8
Sauer	2005		**	**	**	6
Thepcharoennirund	2005		**	–	**	4
Chiu	2006		***	*	**	6
Choudhry	2006		***	–	***	6
Lee	2006		**	*	***	6
A. Li	2006		***	*	***	7
Liem	2006		**	–	**	4
Maksoud-Filho	2006		***	–	**	5
Owen	2006		**	**	**	6
Banieghbal	2007		**	–	***	5
Dutta	2007		**	–	**	4
Menon	2007		**	–	***	5
Riehle	2007		***	–	**	5
Stringer	2007		***	*	**	6
Baglaj	2007		**	*	**	5
Henrich	2007		***	–	*	4
Ishikawa	2008		***	–	**	5
Shinall	2008		***	**	**	7
Spilde	2008		***	*	**	6
Tongsin	2008		***	*	**	6
van Eijck	2008		***	–	**	5
Zheng	2008		***	–	***	6
Dassinger	2009		***	–	**	5
Ferreira	2009		**	–	**	4
Gourlay	2008		***	–	***	6
Hua	2009		***	*	***	7
Liu	2009		***	*	***	7
Obermayr	2008		***	*	***	7
Takahashi	2009		**	–	***	5
Gunnarsdottir	2009		***	**	***	8
Hong	2010		**	*	**	5
Payne	2010		****	*	***	8
Vu	2010		***	*	***	7
de Vos	2011		***	–	***	6
Hill	2011		***	–	**	5
Karimi	2011		***	–	**	5
Kozlov	2010		***	**	**	7
Travassos	2011		***	**	***	8
van der Zee	2011		***	–	***	6
Li	2012		***	–	**	5
Liem	2012		**	*	**	5
Romao	2012		**	*	***	6
Sato	2012		***	*	**	6
Weil	2011		***	*	***	7
Ghaffarpour	2013		***	*	**	6
Ferreira	2013		***	–	***	6
Jensen	2013		***	**	**	7
Nam	2013		***	**	***	8
Nio	2013	2	–	–	–	–
van der Zee	2013		**	–	***	5
Diao	2014		**	*	***	6
Elder	2014		***	–	***	6
Friedmacher	2014		***	**	***	8
Ming	2014		***	*	***	7
Nasr	2014		***	*	***	7
Shrestha	2014		***	–	***	6
Sulkowski	2014		***	**	***	8
Yang	2014		**	–	**	4
Madadi-Sanjani	2015		***	**	**	7
Martinez-Criado	2015		***	**	**	7
Miyano	2015		***	–	***	6
Shangjie	2014		***	*	**	6
Almosallam	2016		***	–	**	5
Diao	2016		**	–	***	5
Guerra	2016		***	*	**	6
Inoue	2016		**	–	***	5
Matsumoto	2016		***	*	**	6
Raitio	2016		***	–	***	6
Diao	2017		**	–	**	4
Dingemann	2017		***	*	***	7
C. Lu	2017		***	–	***	6
Y. Lu	2017		***	*	***	7
Oh	2017		***	**	**	7
Risby	2017		***	–	**	5
Son	2017		***	**	**	7
Tyson	2017		***	*	**	6
van den Eijnden	2017		***	*	***	7
Zani	2017		***	*	***	7
Zmora	2016		***	–	**	5
Avci	2018		***	*	**	6
Peng	2018		***	*	**	6
Xiao	2018	3	–	–	–	–
Zhang	2018		**	*	***	6
England	2012		***	–	***	6
Wakhlu	2000		***	–	**	5
Pratap	2007		***	–	***	6
Abbas	2016		***	*	***	7
Chan	2019		**	*	**	5
de Bie	2019		**	*	***	6
Dewberry	2019		***	**	**	7
Dübbers	2002		***	–	**	5
Gabler	2018		***	–	**	5
Gao	2019		**	–	**	4
He	2016		****	*	***	8
Joda	2019		***	**	***	8
Jona	2001		**	–	**	4
Long	2019		***	**	***	8
Marei	2019		**	*	***	6
Narang	2019		**	*	***	6
Jung	1995		**	–	*	3
Ren	2018		**	*	**	5
Ryu	2019		***	*	**	6
Sakaguchi	2019		***	*	**	6
Sato	1998		***	–	**	5
Sola	2018		***	*	**	6
Yang	2019		***	*	**	6
H. Zhu	2019		**	**	***	7
T. Zhu	2019		***	*	**	6

### Paralytic ileus

In total, 22 studies reported on paralytic ileus and entailed 1,332 patients and 120 events of paralytic ileus [35, 42, 46, 48, 49, 51, 61, 65, 81, 82, 96, 98, 100, 103, 109, 112, 114, 115, 132, 151, 153, 155]. The pooled proportion of total paralytic ileus was 0.07 (95%-CI: 0.05–0.11; *I* ^2^=71%, p≤0.01).

**Figure 2:**
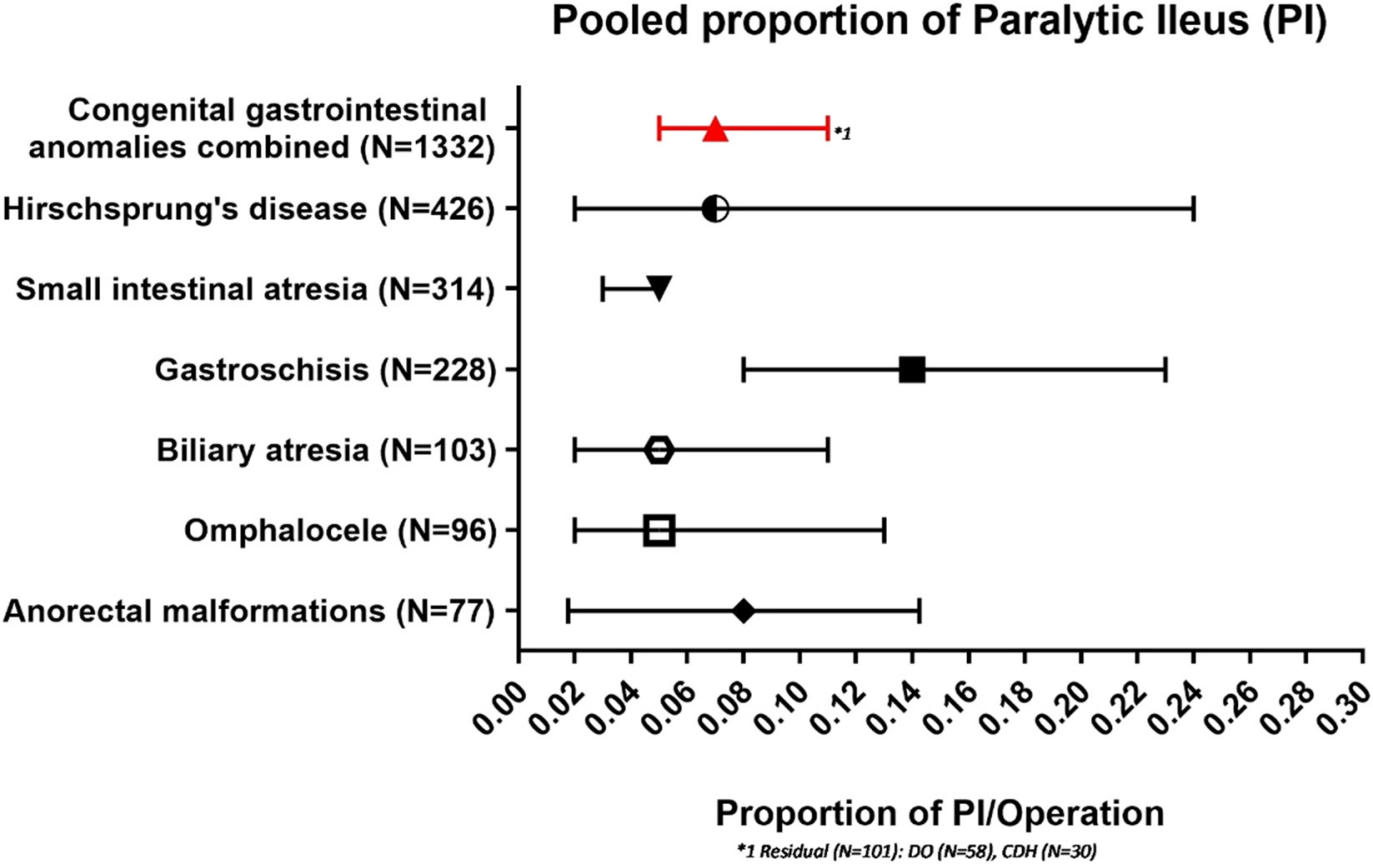
Pooled proportion of paralytic ileus.

Separate pooled proportions were calculated for the following conditions: Hirschsprung’s disease 0.07 (95%-CI: 0.02–0.24; n=45/426; *I* ^2^=91%; p≤0.01); small intestinal atresia 0.05 (95%-CI: 0.03–0.09; n=16/314; *I* ^2^=18%; p=0.25); gastroschisis 0.14 (95%-CI: 0.08–0.23; n=30/228; *I* ^2^=52%; p=0.03); biliary atresia 0.05 (95%-CI: 0.02–0.11; n=5/103; *I* ^2^=0%; p=0.45); omphalocele 0.05 (95%-CI: 0.02–0.13; n=5/96; *I* ^2^=8%; p=0.27); anorectal malformations 0.06 (95%-CI: 0.03–0.15; n=5/77; *I* ^2^=0%; p=0.83). Duodenal obstruction (n=58) and congenital diaphragmatic hernia (n=30) are included in the overall proportion but did not meet the criteria for separate statistical analysis (Figure 2).

### Adhesive small bowel obstruction (SBO)

In total, 99 studies reported on small bowel obstruction entailing 8,470 patients and 572 events of SBO all anomalies combined [10, 11, 14], [15], [16], [17], [18], [19, 21, 24, 26, 29], [30], [31, 33, 34, 37], [38], [39], [40], [41, 43, 45, 47, 49, 51], [52], [53], [54, 56], [57], [58], [59], [60, 62, 63, 67, 70], [71], [72], [73], [74, 79], [80], [81, 85], [86], [87], [88], [89, 92], [93], [94, 96, 97, 99], [100], [101, 103, 105, 106, 109], [110], [111, 113, 115], [116], [117], [118], [119], [120], [121, 123], [124], [125], [126, 128, 131, 132, 134, 136], [137], [138], [139], [140], [141], [142], [143], [144], [145], [146], [147], [148], [149], [150, 152, 154, 156], [157], [158], [159], [160]. Length of follow up was at least half a year in 56 (57%) of the studies.

**Figure 3:**
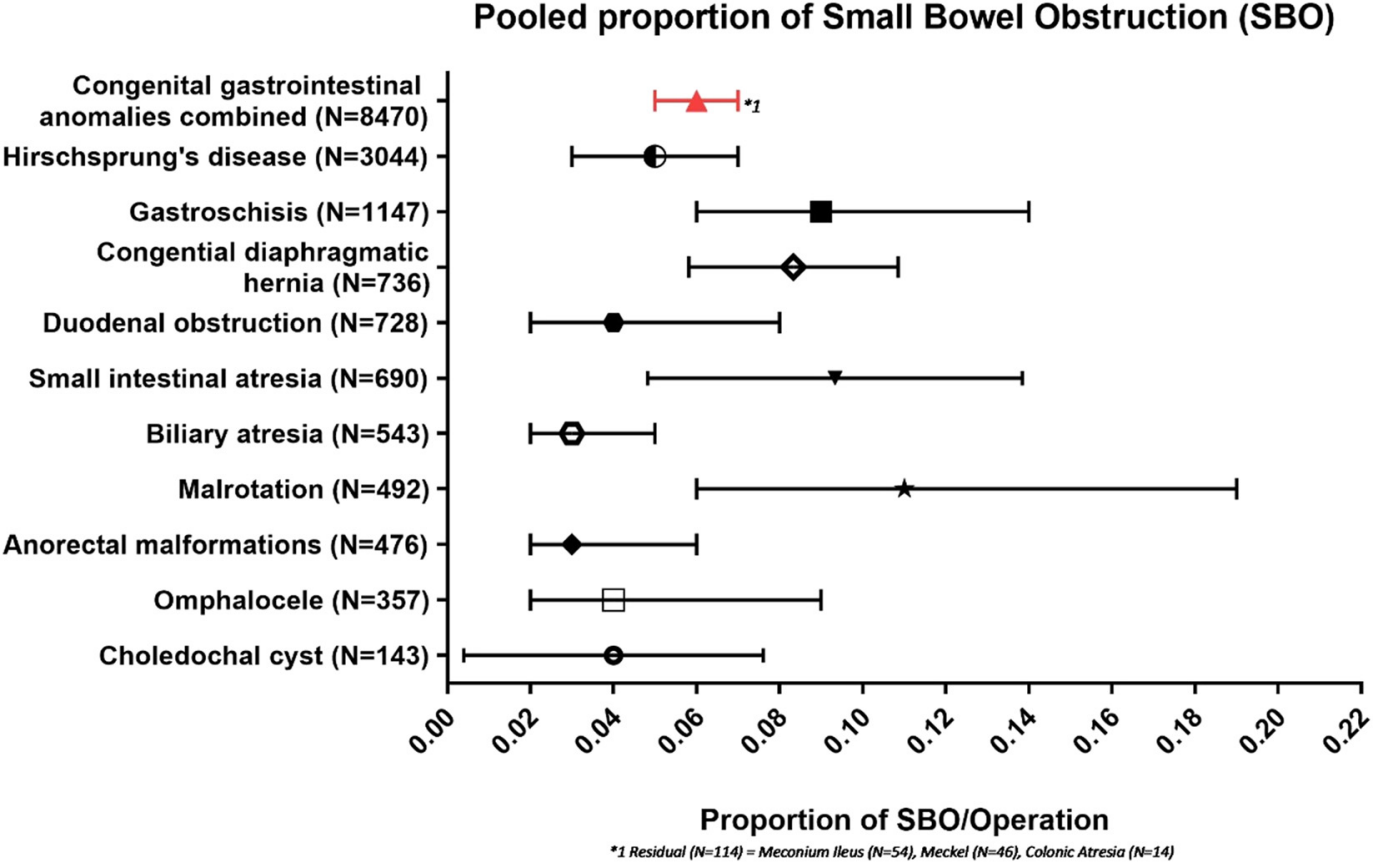
Pooled proportion of small bowel obstruction.

The pooled proportion of total SBO was 0.06 (95%-CI: 0.05–0.07; *I* ^2^=74%, p≤0.01). Separate proportions were calculated for the following conditions: Hirschsprung’s disease 0.05 (95%-CI: 0.03–0.07; n=174/3,044; *I* ^2^=77%; p≤0.01); gastroschisis 0.09 (95%-CI: 0.06–0.14; n=130/1,147; *I* ^2^=75%; p≤0.01); congenital diaphragmatic hernia 0.08 (95%-CI: 0.06–0.11; n=60/736; *I* ^2^=35%; p=0.09); duodenal obstruction 0.04 (95%-CI: 0.02–0.08; n=28/728; *I* ^2^=61%; p=0.01); small intestinal atresia 0.09 (95%-CI: 0.05–0.14; n=60/690; *I* ^2^=74%; p≤0.01); biliary atresia 0.03 (95%-CI: 0.02–0.05; n=19/543; *I* ^2^=0%; p=0.48); malrotation 0.11 (95%-CI: 0.06–0.19; n=46/492; *I* ^2^=73%; p≤0.01); anorectal malformations 0.03 (95%-CI: 0.02–0.06; n=17/476; *I* ^2^=37%; p=0.54); omphalocele 0.04 (95%-CI: 0.02–0.24; n=21/357; *I* ^2^=51%; p=0.31); choledochal cyst 0.03 (95%-CI: 0.01–0.08; n=5/143; *I* ^2^=0%; p=0.48). Meconium ileus (n=54), Meckel’s diverticula (n=46) and colonic atresia (n=14) are included in the overall proportion but did not meet the criteria for separate statistical analysis (Figure 3).

### Anastomotic stenosis

Of the 14 studies reporting on anastomotic stenosis within one month of follow-up, 365 patients were included and 22 events of anastomotic stenosis occurred [13, 18, 20, 35, 57, 59, 64, 75, 78, 108, 109, 118, 129, 151].

The pooled proportion of total anastomotic stenosis within a month was 0.03 (95%-CI: 0.01–0.10; *I* ^2^=81%, p=0.02). Diseases reported on were: small intestinal atresia (n=8/163), Hirschsprung’s disease (n=10/60), duodenal obstruction (n=2/56), anorectal malformations (n=0/30), choledochal cyst (n=0/30) and colonic atresia (n=2/26).

In total, 40 studies reported on anastomotic stenosis after one month of follow-up entailing 4,468 patients and 214 events of anastomotic stenosis occurred [12, 18, 22, 23, 25, 32], [33], [34], [35], [36, 38, 40, 44, 49, 55, 62, 66, 68, 73, 75], [76], [77], [78, 81, 83, 84, 95, 99, 102, 104, 105, 107, 109, 111, 113, 115, 116, 119, 120, 122, 129, 130, 132], [133], [134], [135, 142, 156, 158, 159, 161]. Length of follow up was at least half a year in 29 (73%) of the studies.

**Figure 4:**
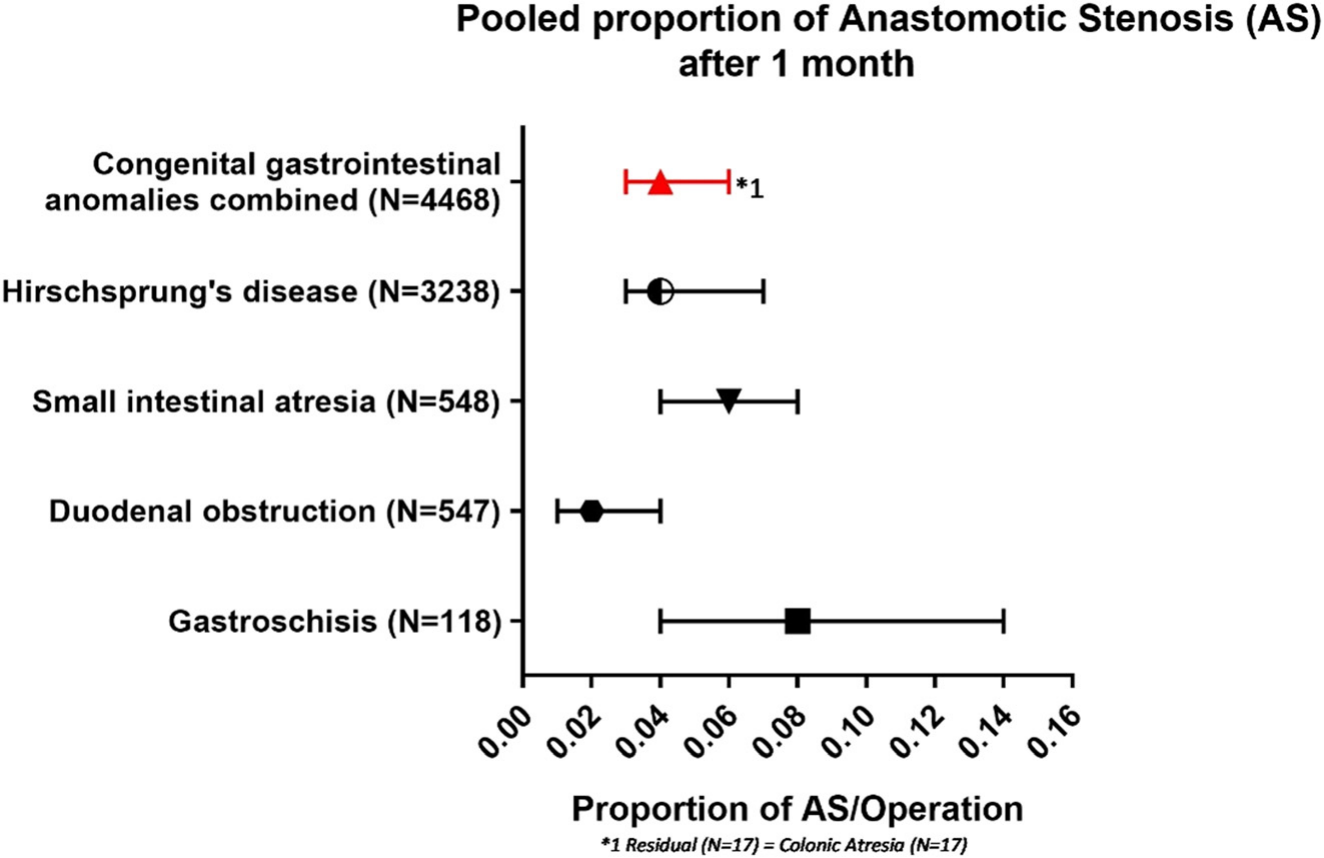
Pooled proportion of anastomotic stenosis after 1 month.

The pooled proportion of anastomotic stenosis was 0.04 (95%-CI: 0.03–0.06; *I* ^2^=59%, p=0.30). Separate proportions were calculated for the following conditions: Hirschsprung’s disease 0.04 (95%-CI: 0.03–0.07; n=162/3,238; *I* ^2^=70%; p=0.11); small intestinal atresia 0.06 (95%-CI: 0.04–0.08; n=32/548; *I* ^2^=0%; p=0.77); duodenal obstruction 0.02 (95%-CI: 0.01–0.04; n=11/547; *I* ^2^=42%; p=0.29); gastroschisis 0.08 (95%-CI: 0.04–0.14; n=9/118; *I* ^2^=0%; p=0.77). Colonic atresia (n=17) is included in the overall proportion but did not meet the criteria for separate statistical analysis (Figure 4).

## Discussion

This systematic review pooled the reported proportions on different types of ileus following abdominal surgery for birth defects in infants. These proportions can be seen as an approximation of the incidences of these complications. According to our reported approximation, these incidences were 7% for paralytic ileus, 6% for adhesive small bowel obstruction, 3% for anastomotic stenosis within one month after surgery and 4% after one month. Within the different birth defects there is a large variation in the occurrence and the spread of these forms of ileus. Although risk factor identification is beyond the scope of this review, the available literature gives some suggestions why these diseases seem to be more at risk.

Out of all diseases paralytic ileus was most common in gastroschisis patients (14%). In these patients, a defect of the abdominal wall leads to extrusion of abdominal content antenatally. Postnatally, this content is reduced intra-abdominally either by primary closure or temporally use of silo and delayed closure. During both procedures the intestine is manipulated severely, which is known to increase the incidence and duration of paralytic ileus in adults [4].

Adhesions, which cause SBO, have long been accepted as a partly inevitable consequence of surgery. They occur as part of the natural healing process. It is hard to define the clinical significance of adhesions, since most are asymptomatic, but when they lead to small bowel obstruction, they can be fatal with mortality rates in children between 2 and 15% [121, 162, 163]. Recently duration of surgery and staged procedures have been identified as risk factors for SBO [37, 162, 164].

Our reported pooled incidence of 6% is comparable to most recent large (n≥100) individual cohort studies reporting on abdominal surgery in infants. These studies report an incidence of SBO between 6 and 10% [37, 165]. It is important to acknowledge that this review entails an aggregated incidence for birth defects only. Acquired diseases such as necrotizing enterocolitis, which seems to be at high risk with a reported incidence of SBO between 25 and 64%, are therefore not included [37, 162].

We found that patients with a malrotation, small intestinal atresia or gastroschisis were relatively most at risk of SBO. This is in concordance with previous studies [37, 121, 162], [163], [164], [165].

We divided anastomotic stenosis into two groups based on reported occurrence within or after one month of surgery since early onset is suggested to be caused by technical error or tissue oedema, whereas a delayed onset and stricture formation is related to chronic inflammation in time leading to anastomotic scarring [166].

Early onset of an anastomotic stenosis is not widely reported and might even be overlooked in the infantile cohort. This review shows that early stenosis does occur and should be considered when conducting research into post-operative complications in the infantile cohort. Technical factors, such as suture reportion speed or mode of suturing, of influence during anastomotic creation should be evaluated to identify risk factors.

Gastroschisis, and to a smaller extent intestinal atresias, were most at risk for late onset anastomotic stenosis. The process of anastomotic healing is to a great extent unclear. Most research has focussed on surgical innovations and techniques without the results leading to a conclusive resolution. Future research in the pathobiology at the cellular level might bring clarification on this matter [166].

This study has its limitations. Because of the variety in study designs and reported outcomes we were not able to look into risk factors which could have lowered heterogeneity. Although it must be noted that, by stratifying for birth defect, some outcomes had moderate to low heterogeneity. Another limitation was that because certain birth defects such as gastroschisis only occur in neonates, our stratification might have resulted in differences in mean age when comparing birth defects. This age difference could be an important reason why certain birth defects are more at risk of certain form of ileus. However, it is not the aim of this review to compare different birth defects but rather report an incidence for each individually. Thus, we believe that this age difference will not hinder the message of our review. If we had only included neonates in this review important birth defects, such as Hirschsprung’s disease, diagnosed beyond the neonatal period would have been excluded. Furthermore, it has to be stated that our results are based on retrospective cohorts available in the literature most of which did not have ileus as a primary outcome. This has undoubtedly increased the chances of occurrence of forms of bias such as selection, publication and reporting bias. Our risk of bias assessment showed most articles to have only fair quality mostly caused by the retrospective, observational nature of most included studies. Moreover, most studies did not have a strict definition of complications possibly resulting in observer bias. Lastly, only 57% of the included articles had a follow-up of at least half a year. Many other articles were unclear about the length of follow up. This lack of long-term follow-up might result in an underestimate of the real incidence of SBO and anastomotic stenosis. SBOs, for instance mostly arise within a year after surgery however episodes are reported 28 years after the initial laparotomy [17, 37, 121, 162], [163], [164], [165]. Although these limitations might have influenced the pooled analyses, at this moment the presented data is the best available approximation of these complications in this cohort.

## Conclusion

This review is the first to aggregate the known literature in order to approximate the incidence of different forms of ileus for each abdominal birth defect. We showed these complications are common and the distribution differs between birth defects. Knowing which birth defects are most at risk might aid clinicians in taking prompt action when an ileus is suspected. Future research should focus on the identification of risk factors and preventative measures. The incidences provided by this review can be used as a starting point for sample size calculations.

## Supplementary Material

Supplementary Material

Supplementary Material
