# Supplementary material for: The incidence of different forms of ileus following surgery for abdominal birth defects in infants: a systematic review with a meta-analysis method
Source: Innov Surg Sci. 2021 Aug 17;6(4):127–50. doi: 10.1515/iss-2020-0042 (PMC9294340; doi:10.1515/iss-2020-0042)
Supplement: Supplementary file 1 — Supplementary Material [file j_iss-2020-0042_suppl_001.docx]

| 3Search block title | Search terms used in PUBMED |
| --- | --- |
| 1. *Age group* | "Infant"[Mesh] OR "Intensive Care, Neonatal"[Mesh] OR "Intensive Care Units, Neonatal"[Mesh] OR "Neonatal Nursing"[Mesh] OR infant*[tiab] OR newborn*[tiab] OR neonat*[tiab] OR prematur*[tiab] OR preterm*[tiab] |
| 1. *Location of surgery* | "Abdomen/surgery"[Mesh] OR "Intestines/surgery"[Mesh] OR "Lower Gastrointestinal Tract/surgery"[Mesh] OR "Upper Gastrointestinal Tract/surgery"[Mesh:NoExp] OR "Duodenum/surgery "[Mesh] OR "Stomach/surgery "[Mesh] OR Abdominal surg*[tiab] OR Abdomen surg*[tiab] OR intestinal surg*[tiab] OR (Lower Gastrointestinal Tract*[tiab] AND surger*[tiab]) OR (Upper Gastrointestinal Tract*[tiab] AND surger*[tiab]) OR Duodenal surg*[tiab] OR Stomach surg*[tiab] OR gastric surg*[tiab] OR "Laparotomy"[Mesh] OR Laparotom*[tiab] OR "Laparoscopy"[Mesh] OR laparoscop*[tiab] |
| 1. *Congenital anomaly* | "Congenital Abnormalities/surgery"[Mesh:NoExp] OR "Abnormalities, Multiple/surgery"[Mesh] OR "Digestive System Abnormalities/surgery"[Mesh] OR "Hernias, Diaphragmatic, Congenital"[Mesh] OR "Urinary Fistula/congenital"[Mesh] OR "Urachus/surgery"[Mesh] OR "Gastroschisis/surgery"[Mesh] OR "Hernia, Umbilical/surgery"[Mesh] OR "Teratoma/surgery"[Mesh] OR diaphragmatic hernia*[tiab] OR Morgagni*[tiab] OR Bochdalek*[tiab] OR ((imperforated anus[tiab] OR anus atresia[tiab] OR anal atresia*[tiab] OR anorectal malformat*[tiab] OR ano-rectal malformat*[tiab] OR intestinal atresi*[tiab] OR duodenal atresi*[tiab] OR jejunal atresi*[tiab] OR ileal atresi*[tiab] OR colonic atresi*[tiab] OR choledochal cyst*[tiab] OR Hirschsprung*[tiab] OR congenital megacolon*[tiab] OR Meckel*[tiab] OR gastroschis*[tiab] OR omphalocel*[tiab] OR urachus[tiab] OR biliary atresi*[tiab] OR teratoma*[tiab] OR teratoid*[tiab] OR dermoid*[tiab]) AND (surgery[tiab] OR surgical[tiab])) OR intestinal malrotation*[tiab] |
| 1. *Post-operative complication* | "Postoperative Complications"[Mesh:NoExp] OR "Anastomotic Leak"[Mesh] OR "Incisional Hernia"[Mesh] OR "Surgical Wound Dehiscence"[Mesh] OR "Surgical Wound Infection"[Mesh] OR "Surgical Procedures, Operative/complications"[Mesh:NoExp] OR "Digestive System Surgical Procedures/complications"[Mesh] OR incisional hernia*[tiab] OR cicatricial hernia*[tiab] OR postoperative hernia*[tiab] OR post-operative hernia*[tiab] OR postoperative ileus[tiab] OR post-operative ileus[tiab] OR anastomotic leak*[tiab] OR anastomosis leak*[tiab] OR stenos*[tiab] OR postoperative periton*[tiab] OR post-operative periton*[tiab] OR postoperative fistula*[tiab] OR post-operative fistula*[tiab] OR complication*[tiab] OR infection*[tiab] OR (adhesi*[tiab] AND obstruct*[tiab]) |

Appendix 1 – Search strategy PUBMED
