# Supplementary material for: The incidence of different forms of ileus following surgery for abdominal birth defects in infants: a systematic review with a meta-analysis method
Source: Innov Surg Sci. 2021 Aug 17;6(4):127–50. doi: 10.1515/iss-2020-0042 (PMC9294340; doi:10.1515/iss-2020-0042)
Supplement: Supplementary file 2 — Supplementary Material [file j_iss-2020-0042_suppl_002.pdf]

Reviewer Assessment

J Derikx, et al.: The incidence of different forms of ileus following surgery for abdominal birth defects in infants: a systematic review with a meta-analysis

Reviewers' Comments to Original Submission

Reviewer 1: anonymous

Date received: 04-Jan-2021
Reviewer recommendation: **Return to author for minor modifications**
Reviewer overall scoring: **Medium**

Assessment Form scores: 5 = High/Yes; 3 = Medium/Adequate; 1 = Low

Is the subject area appropriate for the journal			3		
Does the title clearly reflect the paper's content?			3		
Does the abstract clearly reflect the paper's content			3		
Do the keywords clearly reflect the paper's content?			3		1
Does the introduction present the problem clearly?			3		
Are the results/ conclusions justified?		4			
How comprehensive and up-to-date is the subject matter presented?		4			
How adequate is the data presentation?			3		
Are units and terminology used correctly?		4			
Is the number of cases adequate?		4			
Are the experimental methods/ clinical studies adequate?			3		
Is the length appropriate in relation to the content?			3		
Does the reader get new insights from the article?				2	
Please rate the practical significance.			3		
Please rate the accuracy of methods.		4			
Please rate the statistical evaluation and quality control.			3		
Please rate the appropriateness of the figures and tables.		4			
Please rate the appropriateness of the references.			3		
Please evaluate the writing style and use of language.				2	
Please judge the overall scientific quality of the manuscript.			3		
Are the methods used worthy of reproduction in greater deal?	no				
Would you be willing to review a revision of this manuscript?	yes				

Comments to author: The authors performed a systematic review and meta-analysis evaluating the incidence rates of postoperative ileus after surgical repair of various abdominal birth defects in infants. One-hundred-fifty-two articles (published between 1995-2019) met defined inclusion criteria, reporting a total of 11,617 patients. Pooled proportions for paralytic ileus ranged from 0.05 in omphalocele to 0.14 in gastroschisis, whereas adhesive small bowel obstruction ranged from 0.03 in anorectal malformation to 0.11 in malrotation. The overall rate of anastomotic stenosis less than a month after surgery was 0.03, while anastomotic stenosis rates after a month ranged from 0.02 in duodenal obstruction to 0.08 in gastroschisis. The authors conclude from their analysis that postoperative ileus is a common complication following abdominal surgery and incidence rates differ between the included birth defects.

The authors systematically searched for eligible articles using PubMed and Embase databases. The manuscript draft contains relevant information and was easily comprehensible. However, the study has some ambiguities in its present form:
- The authors state in their abstract that “knowing the incidences of these complications [i.e. different forms of ileus] for each birth defect could aid in developing preventive strategies…”. Which type of strategy/intervention/tool do the authors have in mind that may prevent postoperative ileus following surgery for abdominal birth defects? Please elaborate on that.
- Page 6 line 44 “Only articles that…”. This sentence refers to the inclusion criteria and thus should be mentioned earlier in the methods section.
- Page 7 line 53 “… according to Higgins.” Please provide reference.
- Page 8 lines 23-32: Please provide percentages in addition to the absolute numbers.
- Figures 2-4: There is no need for a legend with different symbols. Simply replace the abbreviation on the left side of the y-axis with the different abdominal birth defects and omit the legend on the right side of the graphs.
- There are several typing, orthographic and grammatical errors, which require revision before considering for publication (e.g. page 4 line 27 “Aadhesive”, line 38 “in order to”, page 7 line 32 “for randomized…”, line 44 “ILEUS”, page 12 line 22 “SILO”, etc.).

Reviewer 2: Stenstrom, Pernilla
Date received: 01-Feb-2021
Reviewer recommendation: **Return to author for major modifications**
Reviewer overall scoring: **High**

Assessment Form scores: 5 = High/Yes; 3 = Medium/Adequate; 1 = Low

Is the subject area appropriate for the journal	5				
Does the title clearly reflect the paper's content?				2	
Does the abstract clearly reflect the paper's content		4			
Do the keywords clearly reflect the paper's content?		4			
Does the introduction present the problem clearly?			3		
Are the results/ conclusions justified?	5				
How comprehensive and up-to-date is the subject matter presented?	5				
How adequate is the data presentation?		4			
Are units and terminology used correctly?			3		
Is the number of cases adequate?		4			
Are the experimental methods/ clinical studies adequate?		4			
Is the length appropriate in relation to the content?	5				
Does the reader get new insights from the article?		4			
Please rate the practical significance.				2	
Please rate the accuracy of methods.			3		
Please rate the statistical evaluation and quality control.		4			

Please rate the appropriateness of the figures and tables.		4			
Please rate the appropriateness of the references.	5				
Please evaluate the writing style and use of language.	5				
Please judge the overall scientific quality of the manuscript.		4			
Are the methods used worthy of reproduction in greater deal?	Yes				
Would you be willing to review a revision of this manuscript?	Yes				

Comments to author: This Thank you for a well written systematic review. Still, some issues here presented are to be further considered.

Titel: Since you do not perform any meta-analysis in terms of comparing two methods, please omit “with a meta-analysis” or write “meta-analysis method”

Abstract: Overall great and clear. Please clarify follow-up time for the outcome regarding the pooled ileus types.

Method
Do I understand it correctly, that the literature searched for, did not need to focus mainly on ileus, and instead ileus could be a result appearing beside the main issue of the studies? This is of importance in judging the reliability of real ileus incidence, especially since the majority were retrospective studies. Please clarify your demand of ileus focusing in the publications included in your analysis.
For anastomotic stenosis ileus, did you define it according to action taken (as surgery or tubes) or was it up on each article's definition? Please clarify this in method.
For follow-up time for complications after one month: did you exclude or include 0-1 month complications? Please clarify it in method and in result.

Results:
Overall the result section is clearly written, easily followed and comprehensive.
Please present the follow-up time for each of the three ileus types of ileus. Also, precise the follow-up time for anastomotic ileus appearing after one month.

Discussion:
For anastomotic ileus within one month, what clinical implications could be of importance with this knowledge in mind?
Please reflect on and elaborate with the quality of the literature included, and if the quality differed between the ileus types reported on.
Please add your thoughts and reflections about how to use this report's reusuts in a clinical setting, or in further research.

Authors’ Response to Reviewer Comments

Date received: 03-Mar-2021

Response to reviewer 1

The authors state in their abstract that “knowing the incidences of these complications [i.e. different forms of ileus] for each birth defect could aid in developing preventive strategies...”. Which type of strategy/intervention/tool do the authors have in mind that may prevent postoperative ileus following surgery for abdominal birth defects? Please elaborate on that.
Reply: We do agree with the reviewer that it would be interesting to discuss preventative strategies, In this light, clinicians could consider early intervention when there is a suspicion of ileus or even preventative measures in these high risk diseases.

Both could be done by nasogastric tube placement, starting treatment with a prokinetic agent such as metoclopramide. Also, it could be warranted to insert a central line during surgery if, due to the ileus, the patient might need trans parenteral feeding. We have now added a paragraph on this in the conclusions of the manuscript and the abstract.
However, the choice of intervention depends on more factors than type of birth defect only. Moreover, these interventions are understudied in young children. For this reason we have not addressed them in this review.
As explained in the limitation paragraph, future research within specific birth defects and diseases should aim to identify risk factors. Depending on the type of risk factors, this could lead to a more fruitful discussion on preventative strategies or interventions. The incidences provided in this research can aid those future studies by providing a starting point for sample size calculations.
We have changed the purpose section of our abstract as to better fit the aim of the review.

Page 6 line 44 "Only articles that…". This sentence refers to the inclusion criteria and thus should be mentioned earlier in the methods section.
We would like to thank the reviewer for this comment and have replaced the sentence so that it is now placed earlier in the method section under Participants.

Page 7 line 53 "… according to Higgins." Please provide reference.
We apologize for this mistake and have added the reference to the text and reference list. In addition we have rechecked our references throughout the manuscript

Page 8 lines 23-32: Please provide percentages in addition to the absolute numbers.
We thank the reviewer for the recommendation. We have added percentages per birth defect.

Figures 2-4: There is no need for a legend with different symbols. Simply replace the abbreviation on the left side of the y-axis with the different abdominal birth defects and omit the legend on the right side of the graphs.
We thank the reviewer for the suggestion and agree that this is a more suitable way to present our results. We have changed the figures as suggested.

There are several typing, orthographic and grammatical errors, which require revision before considering for publication (e.g. page 4 line 27 "Aadhesive", line 38 "in order to", page 7 line 32 "for randomized…", line 44 "ILEUS", page 12 line 22 "SILO", etc.)
Our apologies for these careless mistakes. We have rechecked the whole manuscript and have made the necessary changes. We thank the reviewer for the thoroughness.

Response to reviewer 2

Title: Since you do not perform any meta-analysis in terms of comparing two methods, please omit "with a meta-analysis" or write "meta-analysis method"
Reply: We would like to thank the reviewer and have changed the title from meta-analysis into meta-analysis method.

Abstract: Overall great and clear. Please clarify follow-up time for the outcome regarding the pooled ileus types.
Reply: We have added "of which 86 (56%) had a follow-up of at least half a year" to the result section of the abstract.

Method: Do I understand it correctly, that the literature searched for, did not need to focus mainly on ileus, and instead ileus could be a result appearing beside the main issue of the studies? This is of importance in judging the reliability of real ileus incidence, especially since the majority were retrospective studies. Please clarify your demand of ileus focusing in the publications included in your analysis.

Reply: The number of studies with the primary endpoint of postoperative ileus in infants is very limited. Therefore, in this review we have included al studies mentioning ileus as primary or secondary endpoint. Studies that solely mentioned postoperative complications in general without differentiating between different forms of ileus were excluded. Because we included studies where ileus is not only a primary endpoint and included retrospective cohort studies, underreporting might have influenced the results. Therefore this limitation has been more clearly stated in the limitation section of the discussion section

For anastomotic stenosis ileus, did you define it according to action taken (as surgery or tubes) or was it up on each article's definition? Please clarify this in method.
Reply: We did not include a definition ourselves but included the definition that was used in each article. We searched throughout each article statements on an anastomotic stenosis as opposed to a stenosis without clarifying if the stenosis was based at the sight of anastomosis. We have added extra clarification under primary and secondary outcomes.

For follow-up time for complications after one month: did you exclude or include 0-1 month complications? Please clarify it in method and in result.
Reply: Since we propose that there might be a difference between early (within one month) and late anastomotic stenosis we have evaluated both outcomes separately. This means that we did not include the early stenosis in the late stenosis group which we have now elaborated as well in the method section.

Please present the follow-up time for each of the three ileus types of ileus. Also, precise the follow-up time for anastomotic ileus appearing after one month.
Reply: Due to the methodological differences between studies (some representing mean follow-up duration, others only a range and some did only present the minimum duration of follow-up) we are unable to pool and present the accumulated duration of follow-up. For this reason we decided to count how many articles had a follow up of at least 6 months overall and specifically for the articles describing adhesive small bowel obstruction and anastomotic stenosis. These results are presented in the result section.
In the first draft of our manuscript we evaluated this topic specifically for small bowel obstruction in the discussion section, explaining that this lack of long-term follow-up might have led to an underestimation of the incidence. This lack of long-term follow-up is less of a problem in paralytic ileus and early anastomotic stenosis but could off course also be the case for anastomotic stenosis. In order to make this limitation more clear we have now addressed it in the limitations section.

Discussion: for anastomotic ileus within one month, what clinical implications could be of importance with this knowledge in mind?
Reply: In our view, an anastomotic stenosis that occurs within one month could be the result of the surgical technique used. We believe that technical factors such as suture reabsorption speed, mode of suturing, type of anastomotic creation could influence early postoperative ileus. With the identification of birth defects at risk for postoperative ileus, postoperative strategies might aid in earlier recognition and treatment. As this was insufficiently described in the discussion section we have added our considerations in the discussion section.

Please reflect on and elaborate with the quality of the literature included, and if the quality differed between the ileus types reported on.
Reply: We have re-evaluated our quality scores to assess if there are differences between the different ileus outcomes. We found that articles describing adhesive small bowel obstruction and late anastomotic stenosis on average had 0.5 higher on the NOS compared to early anastomotic stenosis and paralytic ileus. We have added these findings to our results.
The assessment of moderate quality is largely related to the high amount of retrospective, observational studies included in this review. We have added this to the limitation section where we already addressed the moderate quality assessment.

Please add your thoughts and reflections about how to use this report's results in a clinical setting, or in further research.

Reply: Reviewer 1 addressed the same issue in question 1. Summarising our previous answer we think that knowing which birth defects are most at risk might aid clinicians in taking prompt action when an ileus is suspected. Future research should focus on the identification of risk factors and preventative measures. The incidences provided by this review can be used as a starting point for sample size calculations. We have added this to our conclusion.

Reviewers' Comments to Revised Submission

Reviewer 1: anonynous

Date received: 19-Mar-2021
Reviewer recommendation: **Accept in present form**
Reviewer overall scoring: **Medium**

Assessment Form scores: 5 = High/Yes; 3 = Medium/Adequate; 1 = Low

Is the subject area appropriate for the journal			3		
Does the title clearly reflect the paper's content?		4			
Does the abstract clearly reflect the paper's content		4			
Do the keywords clearly reflect the paper's content?		4			
Does the introduction present the problem clearly?			3		
Are the results/ conclusions justified?			3		
How comprehensive and up-to-date is the subject matter presented?			3		
How adequate is the data presentation?			3		
Are units and terminology used correctly?			3		
Is the number of cases adequate?			3		
Are the experimental methods/ clinical studies adequate?			3		
Is the length appropriate in relation to the content?		4			
Does the reader get new insights from the article?			3		
Please rate the practical significance.				2	
Please rate the accuracy of methods.			3		
Please rate the statistical evaluation and quality control.			3		
Please rate the appropriateness of the figures and tables.			3		
Please rate the appropriateness of the references.		4			
Please evaluate the writing style and use of language.			3		
Please judge the overall scientific quality of the manuscript.			3		
Are the methods used worthy of reproduction in greater deal?	Yes				
Would you be willing to review a revision of this manuscript?	Yes				

Comments to author: The authors of this present study have responded sufficiently to the reviewers' comments and revised their manuscript appropriately.

Reviewer 3: anonymous

Date received: 18-Mar-2021
Reviewer recommendation: **Accept in present form**
Reviewer overall scoring: **Excellent**

Assessment Form scores: 5 = High/Yes; 3 = Medium/Adequate; 1 = Low

Is the subject area appropriate for the journal		4			
Does the title clearly reflect the paper's content?	5				
Does the abstract clearly reflect the paper's content	5				
Do the keywords clearly reflect the paper's content?	5				
Does the introduction present the problem clearly?		4			
Are the results/ conclusions justified?	5				
How comprehensive and up-to-date is the subject matter presented?		4			
How adequate is the data presentation?					
Are units and terminology used correctly?	5				
Is the number of cases adequate?	5				
Are the experimental methods/ clinical studies adequate?	5				
Is the length appropriate in relation to the content?	5				
Does the reader get new insights from the article?		4			
Please rate the practical significance.		4			
Please rate the accuracy of methods.		4			
Please rate the statistical evaluation and quality control.	5				
Please rate the appropriateness of the figures and tables.		4			
Please rate the appropriateness of the references.		4			
Please evaluate the writing style and use of language.		4			
Please judge the overall scientific quality of the manuscript.	5				
Are the methods used worthy of reproduction in greater deal?	No				
Would you be willing to review a revision of this manuscript?	No				

Comments to author: The reviewer (#2) thanks the authors for responding to the original comments. Most have been resolved, but I want to comment on two remaining issues, that I believe are not fully resolved.
The authors have provided a clear and concise review on the incidence of different ileus forms after surgery for congenital abdominal defects.
This review has certainly scientific and practical value.

Comments by the Guest-Editor to Revised Submission

The authors clarified all reviewers comment and questions appropriately.
